# Ensuring equitable access, engagement and ability of socially and ethnically diverse participants to benefit from health promotion programmes: a qualitative study with parent carers of disabled children

**DOI:** 10.3389/fpubh.2024.1445879

**Published:** 2024-09-30

**Authors:** Phillip Harniess, Caomhan McGlinchey, Annabel McDonald, Fleur Boyle, Alice Garrood, Stuart Logan, Christopher Morris, Aleksandra J. Borek

**Affiliations:** ^1^Peninsula Childhood Disability Research Unit (PenCRU) and NIHR Applied Research Collaboration South West Peninsula (PenARC), University of Exeter Medical School, University of Exeter, Exeter, United Kingdom; ^2^Nuffield Department of Primary Care Health Sciences, Medical Sciences Division, University of Oxford, Radcliffe Observatory Quarter, Oxford, United Kingdom

**Keywords:** behaviour change interventions, health inequality, wellbeing, caregivers, implementation science, interviews, social determinants of health, community interventions

## Abstract

**Background:**

Equity is fundamental to health promotion programmes. However, unintentional or unseen barriers may exist for some underserved groups. We aimed to identify how to ensure equitable access and engagement for diverse parent carers of disabled children to benefit from health promotion programmes.

**Methods:**

We purposively sampled parent carers with potentially intersecting characteristics including those who self-identified as from ethnic groups, whose children were educated other than at school, with sensory impairments, or neurodiversity, and fathers. Participants were recruited through local and national organisations and parent carer networks. Data collection involved semi-structured individual interviews, which were transcribed verbatim and analysed thematically and iteratively alongside data collection. Core researchers performed early analysis independently, followed by research team and advisory group cross-validation.

**Results:**

Thirty-six parent carers with intersecting characteristics across the sampled backgrounds participated. We identified various perceived barriers around finding out about, attending and engaging with health programmes. We organised the findings into five themes focused on concepts capturing challenges and potential solutions to contextual barriers to access and participation in health programmes. (i) Reach—judiciously using targeted and universal strategies to ensure equitable distribution; (ii) Credibility—demonstrating trustworthiness of those advertising and/or delivering the programme; (iii) Opportunity—ensuring that the programme is seen as fulfilling a relevant need; (iv) Reservations—addressing barriers of readiness to participate; and (v) Optimisation—tailoring to improve the inclusivity of the programme delivery.

**Conclusion:**

We identified modifiable factors that impede members of some social groups from engaging with, and benefiting from, health promotion programmes, and potential solutions. We advocate a multifaceted approach is required from outreach to delivery, tailored to be mindful of extant diverse needs of parent carers in underserved communities. We catalogue key considerations to inform implementation strategies to optimise equity in health programmes for parent carers. The implications are likely transferable to other interventions and contexts.

## Introduction

Public health promotion interventions are *“planned actions to prevent or reduce a particular health problem, or the determinants of the problem, in a defined population*,” [p. 520, (1)] and so require intentionality in their design and implementation. Despite good intentions, there is a risk that these interventions might reproduce or worsen inequalities by primarily favouring individuals with greater advantages (2). Therefore, investigating how interpersonal (e.g., family), social (e.g., ethnicity) and wider contextual factors influence health behaviour change interventions is critical to informing programme design and delivery to promote equity (3–6). Accessibility and acceptability may also be unequal for some groups compared to others, which has implications for participant recruitment and intervention evaluation (4, 7, 8). Some health promotion and behaviour change interventions have shown promise at reducing inequity through contextual adaptation at individual and community levels with different groups (6, 9, 10). However, there are notable research gaps; (i) in understanding how to reach and engage underserved groups in health research (7, 8) and; (ii) in accounting for how social determinants influence interventions and outcomes across different social contexts (6).

The concept of health equity has been defined as the “highest level of health for all people,” “opportunity for all,” and “absence of disparities” (11). Proportionate universalism proposes that “*greater intensity of action is likely to be needed for those with greater social and economic disadvantage*” [p. 16, (5)]. Descriptions of populations with greater social and economic disadvantage have drawn debate. Descriptors such as “underserved” or “equity deserving,” as opposed to “vulnerable” or “equity-seeking,” underline the principle that marginalised groups deserve health equity as a right, and “seeking” should not be their burden (12). We use “underserved,” where it is defined as:

“*A group that is less well represented in research than would be desirable from population prevalence and healthcare burden*.” (13)

In comparison to other parents, parent carers of disabled children are more likely to experience compromised physical and mental health and wellbeing (14–17). Additionally, parent carers are more likely to derive from disadvantaged groups (18, 19). While arguably all parent carers have underserved health needs, there may be structural inequalities affecting some groups more than others, which are associated with higher risk (18–21). These inequalities map broadly onto well-established social determinants of population health (5), whereby personal, psychosocial and economic factors interact to shape and exacerbate health and wellbeing problems (22). Marmot (5), indicates that “*these factors are influenced by social position, itself shaped by education, occupation, income, gender, ethnicity and race*” (p. 16).

A health and wellbeing promotion programme uses systematic design and actions to empower individuals and communities to address health determinants with the aim of improving health and wellbeing (23). Henceforth we will refer simply to “health programmes.” A range of parent carer-focused health programmes have been developed to improve health outcomes for parent carers and their children (24–27). Although different in their design and approach, they have a shared challenge of reaching underserved parent carers who would benefit from such interventions. We co-created Healthy Parent Carers (HPC) as a community-based programme that aims to improve the health and wellbeing of parents of disabled children (Box 1) (24, 28–30). The HPC programme is predominantly delivered by third-sector organisations on behalf of Local Authorities. Third-sector organisations refer to those outside of the state or market, incorporating diverse groups such as charities, nongovernmental organisations and social enterprises (31). Within the UK, third-sector organisations are often commissioned to deliver health programmes.

**BOX 1 Overview of HPC programme.**

**Background**:

The Healthy Parent Carers (HPC) programme aims to promote parent carers’ engagement with health promoting behaviours to improve resilience and overall health and wellbeing.

Co-created by parent carers and researchers, it is based upon behaviour change techniques (e.g., goal setting) and a set of universal and evidence-based actions associated with health and wellbeing: CLANGERS, which stand for Connect, Learn, be Active, take Notice, Give, Eat well, Relax and Sleep, an extension of “the five ways to wellbeing” (28).

**Delivery**:

- Groups consist of 6–12 parent carers
- In-person or online via videoconferencing (e.g., Zoom)
- Trained Lead and Assistant Facilitators, who are also parent carers, deliver the programme
- 12 modules with two-hour sessions covering CLANGERS content and behaviour change techniques, such as goal setting and self-monitoring
- Facilitated group-based activities utilising health-related information and resources, via printed or online materials and videos

**Setting**:

To date UK based

Spans different geographical regions from urban to more rural

**Summary of programme theory**:

The peer-led group-based programme is intended to facilitate change by providing opportunities for and prompting: social and emotional (peer) support, valuing the shared social identity as parent carers who want to look after their health, sharing experiences, and embedding practice of health-promoting behaviours.

More details, including the intervention and implementation logic models, are published elsewhere (24, 28, 30).

The aim of our study was to identify how to help ensure equitable access and engagement for socially and ethnically diverse parents of disabled children to benefit from parent carer-focused health programmes.

Regarding terminology, we acknowledge debate around differing preferences for referring to “disabled children” or “children with disability.” We use “disabled children,” preferred by advocates of the social model of disability, because the child/person is disabled by society or the environment (32, 33). “Parent carer” is a UK legal term derived from the Children and Families Act (2014) to describe parents who are primary adult caregivers of disabled children and young people up to 25 years (34). These children and young people are assigned Special Educational Needs and Disability (SEND) rights for inclusive education (35). We have adopted the term “Global Majority” as a more appropriate descriptor of persons often labelled as “ethnic minorities” as this term challenges “*the debilitating implications of being racialised as minorities*” through a Western normative lens [p. 22, (36)]. Further, we were conscious to avoid applying “master” categories of social position, such as race/ethnicity, to be proxy for other characteristics, such as poverty or educational status. Intersectionality considers how various aspects of one’s identity (e.g., race, gender or educational status) interact to create unique experiences of privilege or disadvantage (37) that may also compound barriers to health interventions, such as those relevant for parent carers in the context of this study. Therefore, our study sought to understand the context of inequity in parent carers’ lives relating to their health and wellbeing and access to programmes in all its complexity, through intersectionality (38).

## Methods and materials

### Public and other stakeholder involvement

We carried out Patient and Public Involvement (PPI) through our Family Faculty group facilitated by a Family Involvement Co-ordinator (AMcD) who is a parent carer. The Family Faculty is made up of families of disabled children in the UK who are interested and involved in work carried out by the research group (39). For this study, we convened a study-specific PPI working group with volunteers from members of the Family Faculty from diverse backgrounds, many of whom had experience of the HPC programme through its co-design process, or as attendees. The PPI group advised on all stages of the research. The Guidance for Reporting Involvement of Patients and the Public (GRIPP-2) short form (40) is used to describe the collaboration with our PPI group for this study (Table 1).

**Table 1:** Public and patient involvement in this research described using the GRIPP-2 short form.

Topic	Item
Aims Report the aim of PPI in the study	The diverse group of parent carer PPI contributors were encouraged to bring their lived experience and expertise with the aims to: Provide a focus on the most meaningful aspects/factors of equity in parent carer-focused health programmes Contribute to the study sampling approach by identifying and prioritising target groups of parent carers who might experience inequity Provide advice and practical support for the recruitment strategy, e.g., access to diverse parent carer networks Pilot and feedback on the interview topic guide Challenge the researchers’ implicit biases and assumptions with a particular emphasis on the interpretation of findings Consider the implications of findings for implementation strategies locally and for broader research/practice in this field Support sharing of findings Further stakeholder meetings were held with HPC programme facilitators to further consider the implications of the findings for programme delivery and implementation.
Methods Provide a clear description of the methods used for PPI in the study	Over the course of the study 10 parent carers were involved in the PPI group meetings, six of whom had previous experience of the HPC programme as participants, one was a trained facilitator; and the others did not have direct experience of HPC. PPI group members were from diverse ethnic backgrounds and lived in different urban and more rural settings across the UK; one member was a father. Group meetings were co-designed and co-facilitated between two researchers and the Family Involvement Coordinator (who is also a parent carer) to support input from parent carers relating to all aspects of the study. Five group online meetings were convened and focused around the stated aims: Consensus discussions supported the prioritisation of target groups and sampling approach. Parents were encouraged to advise and provide support connecting the researchers to other organisations and networks of target underserved parent carers groups. We followed up individually with those who offered to support around this activity. One PPI group member went further by adopting a chaperone role to parent carers from a Muslim background in three interviews. The interview topic guide was piloted and cognitively tested during one PPI meeting and feedback was given specifically relating to wording and interpretation of questions and areas for potential further probing. As data began to be collected, emerging analysis was shared with the group for discussion through vignettes to sense-check and challenge the assumptions of the researchers to build a more robust representation and interpretation of the data. PPI members supported the strategy around the application of the findings into practice, for example suggesting key stakeholders to involve in the next stage of implementation. PPI members were also involved outside of the group meetings in reviewing study documents, such as the participant information sheet, study advert and consent form and later in the development of the plain English summary of the findings for wider sharing. Two further stakeholder meetings were held with 27 HPC programme facilitators enabling further consideration of the implications of the findings for programme delivery and implementation. Parallel engagement work was undertaken to support our awareness and the recruitment strategy, involving key organisations: The Fatherhood Institute, DadMatters, Look UK, Tower Hamlets Opportunity Group, The Esteem Team.
Results Outcomes—report the results of PPI in the study, including both positive and negative outcome	PPI strengthened the study in multiple ways: Directly enabled recruitment of parents from underserved communities, e.g., one member, a British Pakistani mother, became a gatekeeper inviting eight potential participants, five of whom decided to take part. A PPI group member chaperoned for Muslim mothers, where requested, and directly supported some parents with low digital literacy to access the video conference call for interviews. The PPI group advised the researchers directly around cultural sensitivities when approaching and engaging with members of their ethnic community. Other groups, e.g., charities provided similar strategic advice relating to recruitment. The study topic guide was developed with the PPI group, who advised on the use of language and tested it through pilot group interviews. The data analysis was presented and discussed with the PPI group acting as critical friends. Overall, they reported a resonance with the findings and felt they were sensitively developed. They provided direct advice on the relevance of the findings and implementation ideas for parent carer health programmes and HPC specifically.
Discussion and conclusions Outcomes—Comment on the extent to which PPI influenced the study overall	As discussed above, the PPI had a positive and direct influence on the study; specifically, it supported success around recruitment, the collection of rich data and the development of credible and resonant findings and identification of practical implications.
Reflections/critical perspective Comment critically on the study, reflecting on the things that went well and those that did not, so others can learn from this experience	The data collection via interviews occurred online for feasibility and pragmatic reasons. When engaging with different communities, a multipronged approach was used to build trust and relationships. However, we had limitations on being able to go to meet with different groups face-to-face. Meeting face-to-face may have optimised relationship building in certain communities and the participation of more underserved parent carers. Nevertheless, our strategy did still enable effective involvement of many parent carers who we would not have been able to access otherwise. Further engagement work is needed with the PPI group to build on the implementation strategy and its application into practice.

In addition, we consulted with two groups of facilitators of the HPC programme regarding the findings. This enabled further sense-checking and an immediate opportunity to consider the delivery and implementation strategies.

### Participant sampling

All participants were parent carers of disabled children living in the UK. We used purposive maximum variation sampling for parent carers across different underserved groups who had experience of the HPC programme (as facilitators or participants) or no experience. Sampling was informed by our PPI group which itself included parent carers from diverse backgrounds, as well as by UK national guidance on reducing healthcare inequalities (41). We focused on groups who would provide insight into different aspects of inequity in this area, including: ethnically diverse, fathers, parents with sensory impairments and parents whose children are educated at home. This latter group includes parents of children Educated Other Than At School (EOTAS) (42) and those who are Electively Home Educated (EHE) (43). We recorded other relevant intersecting characteristics, including neurodiversity, Indices of Multiple Deprivation (44), educational status, single/co-parenting status and child history. While we were cognisant of intersectionality as important in equity research (45), sampling of all potentially possible intersectional permutations was unfeasible. As we analysed data and recruited participants concurrently, we identified specific groups who continued to provide novel insights even after our initial analysis, akin to theoretical sampling (46). Therefore, we sampled proportionately more participants from ethnically diverse backgrounds.

### Recruitment strategy

Our recruitment strategy was multifaceted. We posted adverts on social media platforms with invitations including a link to the study website with participant information and an online expression of interest form. The expression of interest form asked potential participants about their characteristics to allow for purposive sampling. All potential participants who had expressed their interest were contacted by telephone or e-mail and screened. Eligibility was assessed against the basic parent carer information and the fit with the target sample groups. We informed those who were ineligible by email or phone.

To ensure recruitment from underserved groups, we took additional steps. Ethnically diverse members of our PPI group approached parent carers within their communities about the study. We collaborated with local and national third-sector organisations for the target sample groups, who shared the study adverts across their networks. In four cases during the recruitment process, prospective participants were offered a chaperone while being interviewed. The offer of a chaperone is considered respectful in many communities, and those who elect to have chaperones feel more relaxed, supported, and safe (47). In cases where this offer was accepted, a chaperone was identified. In three cases the chaperone was a female member of our PPI group, she was from a Pakistani background and was familiar to the participants; in the fourth case, a representative from a national charity was requested by the parent carer with a visual impairment to support them as a chaperone because they had a strong pre-existing working relationship.

All participants provided consent and received a £35 voucher following interview.

### Data collection

We conducted one-to-one semi-structured in-depth interviews via video conference calls from September 2023 to February 2024. Interviews were conducted by one of two researchers (CMcG/PH) and lasted approximately one hour. A semi-structured topic guide was created and piloted with the PPI group (Supplementary Document). It included open questions, followed by more specific prompts, about accessing and engaging with health programmes. Within the framing of their social identity (e.g., “as a father …”), we encouraged participants to reflect on factors that enabled or impeded their access and engagement with such programmes. Interviewees who had attended the HPC programme were asked to reflect on these factors both before and after joining the course and facilitators in relation to its delivery. Interviewees who had no experience of HPC were asked to consider factors relevant to them and their community when thinking about other hypothetical or experienced parent-carer programmes. Interviews were audio-recorded and transcribed verbatim.

### Data analysis

We used reflexive thematic analysis methods (48). Our process involved recording written summaries of each interview, which we used during weekly team discussions alongside data collection to enable a responsive approach to subsequent recruitment, interview probing and analysis. Verbatim transcripts were uploaded to NVivo v.14 software for coding. To develop a codebook, three researchers independently coded the same sub-sample of transcripts (CMcG and PH *n* = 5 each, AB *n* = 2). Each researcher arranged the codes into candidate categories. The categories and codes were compared and discussed and integrated into one combined codebook. It was then used by CMcG and PH in subsequent analyses of transcripts. Where new and relevant phenomena were observed, additional discussions supported the further development of the coding framework. Findings were regularly reviewed with the research team and periodically with the study PPI and stakeholder groups.

The researchers conducting the analysis included two White British males and a White European female from diverse professional and academic backgrounds (educational psychology, physiotherapy and sociology). To remain cognisant of our positionality during interviews and within the analysis and ensure rigour, we used reflexive practices such as writing memos, (peer) supervision and discussing differences in coding interpretations. In addition, triangulation was vital with the PPI and stakeholder groups, which included those from underserved groups, to increase the validity of findings (49, 50).

### Ethics approval

The study procedures were approved by the University of Exeter Medical School Research Ethics Committee (UEMS REC ID: 525009).

## Results

Thirty-six parent carers were interviewed; 20 had never attended a parent carer-focused health programme, 10 had previously attended the HPC programme, and six were Lead or Assistant Facilitators on the HPC programme. There were 16 from Global Majority groups, 12 were fathers, seven were parents of children who were EOTAS and four parents had sensory impairment (Table 2).

**Table 2:** Participant characteristics (*n* = 36).

		*N*	%
Purposively sampled characteristics	Global Majority background	16	44.4
	EOTAS or EHE*	7	19.4
	Fathers	12	33.3
	Sensory impairment	4	11.1
Additional relevant characteristic	Neurodiverse (ASC/ADHD**)	4	11.1
Parent carer mean age—years (Range)		47 (33–60)	
Parent carer sex	Female	24	66.7
	Male	12	33.3
Partner at home	Yes	23	63.9
	No	13	36.1
Parent education	Degree or above	27	75.0
	Educated to 19 years old	3	8.3
	Educated to 16 or below	6	16.7
Child’s main diagnosis	ASC/ADHD*	31	86.0
	Learning disability	4	9.7
	Physical neurodisability (e.g., cerebral palsy)	6	13.0
	Visual or hearing impairment	5	12.2
Mean child age—years (Range)		12.3 (3–20)	
Deprivation in area of residence (Indices of multiple deprivation***—IMD; quintiles)	1 (most deprived)	5	15.1
	2	15	45.4
	3	3	9.0
	4	3	9.0
	5 (least deprived)	7	21.2

* Educated Other Than At School (EOTAS) | Electively Home Educated (EHE).

** Autism Spectrum Condition (ASC)/Attention Deficit Hyperactivity Disorder (ADHD).

*** English Indices of Multiple Deprivation 2019 (44).

We constructed five themes relevant to enhancing equity in access and engagement with parent carer-focused health programmes, which focused on the central concepts: “Reach,” “Credibility,” “Opportunity,” “Reservations” and “Optimisation” (Table 3). We elucidate upon each theme using supporting quotes, attributed with initials to indicate whether the participants were HPC programme attendees (A), non-attendees (NA) or facilitators (F) and other relevant characteristics. Our findings are presented for the whole sample and, where relevant, we note barriers that linked with specific characteristics (e.g., ethnicity, sensory impairment).

**Table 3:** Barriers and strategies to enable participation of underserved parent carer groups in health programmes.

Barriers	Strategies
Reach: To effectively reach underserved populations, particularly those outside of mainstream networks which hinders access to information and support	Make efforts to understand and address the needs of diverse communities to ensure an equitable reach for underserved groups. Make intentional efforts to communicate with underserved populations who may not be connected to or have awareness of existing networks or places where health programmes are advertised, i.e., use targeted outreach efforts—“Going to where they are.” Partner with representative and trusted community organisations that have existing contacts or can develop new relationships within specific communities to deliver the message. Address language and cultural barriers that prevent parent carers from accessing information. Recognise the importance of engaging both mothers and fathers and seek appropriate ways to reach and engage both mothers and fathers. Use both universal and targeted advertising strategies for the programme.
Credibility: To establish trustworthiness so that the message is not dismissed. Mistrust towards providers or organizations can lead to message dismissal.	Recognise the importance of credibility in messaging about health programmes, understanding that mistrust towards messengers or organisations can lead to dismissal of the message. Build trustworthiness through a credible track record of community engagement and by demonstrating an understanding of different community needs. Work with and through trusted community partners and local organizations to promote programmes, tapping into the community’s existing networks. Provide advocacy and practical support to parent carers, demonstrating a commitment to their wellbeing beyond programme promotion. Engage in relationship building by spending time with ethnic communities and understanding their specific needs and cultural nuances. Utilise word-of-mouth recommendations and testimonials, especially from peers within underserved groups with firsthand experience of the programme to enhance credibility. Include representative images of parent carers from the target audience in programme advertisements to increase relatability and trust.
Opportunity: There may be barriers to different groups’ perceptions that a programme meets a relevant need	Understand that the perception of need might be constrained by sociocultural norms, language, and stigma around seeking support and sensitively address these through peer interaction and tailored messaging. Present a clear, resonant and appealing message to the target audience, e.g., sensitive use of language, accessible and culturally appropriate advertising materials. Consider language translations and the choice of wording to make the opportunity more inclusive Avoid using generic terms like “parent” and instead use “mums and dads.” Use pictures or videos with fewer words to be more inclusive of parents with dyslexia or those less confident in English. Work with underserved communities to highlight the evidential risks to health and mental wellbeing for parent carers. Emphasise benefits and attractiveness of the programme to ensure it is perceived as relevant and beneficial.
Reservations: There may be barriers to participating in the programme, including readiness to engage, and social, practical and emotional factors	Acknowledge reservations stemming from social anxiety, low confidence, and a sense of isolation among parent carers, which may be greater for underserved groups. Appreciate that acceptance of the parent carer identity may influence openness to joining peer programmes and sense of belonging within the group. Create opportunities to meet other parent carers by providing comfortable spaces as a social entrance point, e.g., coffee mornings or groups for practical learning, particularly for fathers Prioritise pre-programme engagement conversations to build rapport, cultivate readiness, address socioemotional anxieties, assess needs and plan adjustments. Address concerns about gender balance within groups and perceptions of fathers’ roles Work with the strengths of existing belief structures of ethnic communities and empower parent carer awareness of health and wellbeing needs. Address practical considerations, such as timing, childcare, and transportation, especially for hesitant underserved groups. Recognise how social circumstances, such as overwhelming caregiving responsibilities and crises, may affect a parents’ capacity to be ready to participate in a programme. It may be more appropriate for some parents to participate in a future programme.
Optimisation: There may be barriers in the inclusivity, acceptability and effectiveness of a programme according to its sensitivity to language, sociocultural norms and mode of delivery	Understand that mixed-gender or single-gender groups have different implications for attendance and openness and may appeal to different groups. Cultivate a shared social identity around the core common experience(s) of being a parent carer, where attendees feel comfortable regardless of other differences. Appreciate subtle cultural expectations and preferences, such as communication styles and hospitality. Address the needs of parent carers with sensory impairments or neurodiversity by ensuring materials and activities are accessible. Provide translations of programme materials, incorporating cultural adaptations where necessary. Consider both online and in-person delivery to accommodate different barriers related to attendance.

### Reach: judiciously using targeted and universal strategies to ensure equitable distribution

A primary challenge acknowledged across groups, and core to health inequity, was reaching those less or not engaged with health programmes, rather than “*engaging with people who are already engaged*” (A3, British Asian, father). “Reach” accounts for the intentional and informed effort to communicate with underserved populations because it is recognised that some parent carers are not connected into networks and places where programmes might be advertised.

*“… people who are not part of any of the networks … how likely they are to sign up for it. I do not know.” (F36, British Bengali mother)*

In more patriarchal cultures, it was explained that “*you need to convince the husband as well*” (NA20, Asian mother). Particularly where English was a second language for the mother, fathers were more likely to source relevant information (e.g., via school or the local mosque/masjid) and pass it on to their partners.

Participants, in reference to their group, highlighted that it was commonplace that there were parents unfamiliar with the “system” who do not know where to look or how to ask for support, including such health programmes.

*“… how are people supposed to get information? … how do they know where to go in the first place though? [Laughs] … the demographics of this area, there’s people that do not know the system, they do not speak the language, there’s no advocacy services.” (NA14, British Pakistani mother)*

This scenario might represent an inequity in SEND cultural capital, whereby leveraging support for their child and family is constrained, and a barrier to finding out about parent carer-focused programmes.

Participants discussed contrasting universal or targeted advertising strategies. A universal approach taps into existing statutory structures and services with “*blanket*” coverage and contact with parent carers in places, such as Special Educational Needs schools, children’s centres and the health sector. It was seen to have comprehensive reach. In particular “*the diagnostic services*” for the child (e.g., paediatricians and child development centres) were identified as potentially strategic distributors because “*that’s when parents are seeking help at that point*.” One parent argued that going through statutory bodies was most equitable because it avoided the “*inconsistent*,” “*disjointed*” and “*patchwork*” coverage of third sector organisations. Social media was also advocated for as an advertising strategy.

However, this universal strategy was seen as less effective at reaching underserved groups. Single fathers reported being excluded from their child’s Special Educational Needs (SEN) school email lists, while parents of EOTAS children stated that they “*end up disappearing*” by no longer having access to conventional information shared through schools. Organisations, such as local authorities, that might control distribution were criticised as “*sending emails blindly*” to mailing lists that were inherently exclusive, while simultaneously not working hard enough to understand all their populations’ needs. Their ineffectiveness at reaching underserved groups was contrasted to a more effective targeted strategy and making efforts to build relationships and understand different communities’ needs.

*“I guess it’s going to where they [underserved parent carers] are, rather than them coming to us … like I said, with the Somali community, we are going out to them … rather than us just saying, ‘yeah, the flyer and whoever comes’ we will have to go out there and we’ll have to package it differently.” (F2, British Bengali mother)*

“*Going to where they are*” was widely reflected as an essential principle. The targeted approach largely depended on providers within the third sector who were representative and/or knowledgeable of their community, with existing contacts. Or it depended on those who were able to develop new relationships with groups to gain access to influential organisations/institutions within specific communities to deliver the message (e.g., community [Somali] group coffee mornings, visual impairment charities, EOTAS support networks, fathers’ groups, mosques/masjids). Overall, diverse and informed universal and targeted strategies were advocated to ensure an equitable reach for underserved groups.

### Credibility: establishing trustworthiness of those advertising and/or providing health programmes

Even when parent carers find out about a health programme, participants highlighted that the credibility of the person/organisation (“messenger”) was essential. In some cases, this was the programme provider and in others a signposting organisation, such as the Local Authority. Mistrust towards individuals or organisations providing the message, caused by previous negative interactions, was suggested to create a risk that the “message” would be immediately dismissed.

*“… with their association … they have had a bad experience with this person or that person [*e.g., *a local authority], and they think this is just an extension to that and it’s going to be a similar negative experience…” (A3, British Asian father).*

Evidently, the trustworthiness of the messenger was key for people to give initial serious consideration to the message. Such trustworthiness was suggested to be created through a credible track record of authenticity in serving and working in partnership with groups in the local community, with an informed understanding of their culture and needs.

*“There’s a local organisation, they do health and wellbeing sessions for children, families, carers, the whole community … if they were promoting such a programme, parents have got trust in the [organisation], so the [organisation] would not promote something that would not be in their best interest. It’s about tapping into the local community, and looking at trusted partners that you can work alongside in order to publicise, more than it just being an ad on the internet.” (NA11, Black Caribbean British mother)*

It was critical how parent carers perceived the intentions that those advertising and providing the programme had for offering it. Best practices were shared of how facilitators within organisations providing the parent carer programme had worked in ways to also become trusted local providers. For example, by providing advocacy and practical support in relation to their child’s SEND rights in education or meeting other social needs. Hence, when they informed parent carers about an opportunity, it was received positively and was perceived to be an extension of their support work. Other providers understood similar contextual barriers and engaged in relationship building by spending time with ethnic communities in existing gatherings in order “*to let the parents know exactly what the programme is about so they can sign up if they are interested*” (F28, Somali mother). Proactive providers also offered events sensitive and appealing to local cultural groups with food and hospitality to draw together those more isolated parent carers, which created opportunities for introduction to the parent carer network. Accordingly, bringing groups of parent carers together was often a foundational precursor to offering programmes.

Another feature which participants emphasised to enhance message credibility was “word of mouth” recommendations. This approach was particularly powerful when imparted by a peer known to the parent, who held an inherent shared empathy with first-hand experience of the programme.

*“… because it was recommended by [parent carer from her community] … I know … she is a really intelligent lady … Whatever she’s doing, it must be very good … So I think I would go for it.” (NA13, Pakistani mother)*

In some situations, previous attendees from particular underserved groups, were tasked with providing honest testimonials to their peers, e.g., via the SEN school. Similarly, adverts with testimonials, including pictures of representative parent carers of the target underserved group (e.g., fathers) was seen as a subtle way to enable resonance, because seeing “people like me” indicated that this programme could be trusted and equally be “relevant for me.”

### Opportunity: ensuring that the programme is seen as fulfilling a relevant need

Further potential barriers to access and engagement related to how the opportunity of attending the programme was presented. Participants indicated that the message needed to be clear, resonant and relevant for them individually and within their social group. Therefore, the design of advertising was influential to whether people would perceive an opportunity as relevant and inclusive. Language translations were recommended, and also consideration in the choice of wording was essential. For example, fathers frequently expressed that many would not consider themselves as the primary caregiver and would defer to their partner as the “parent carer,” unless the father was a single parent carer, or the established primary caregiver in a couple. Therefore, fathers recommended that the generic “parent” term be avoided when advertising the programme.

*“If you then go ‘mum and dad’, the word ‘dad’ [is] there so it’s not like it feels so exclusive.” (A4, White British father, EOTAS)*

Using pictures (and fewer words) or videos was suggested as it can be more inclusive of parents with dyslexia or those less confident English speakers.

Many participants articulated a complex barrier regarding whether they might engage with a programme based upon their sense of self-identity. Participants highlighted how social norms and their social identity could constrain identification of need and support seeking particularly for fathers and some ethnic groups. For some, the “parent carer” term was not familiar as it related to the “*SEND world*” to which they felt unconnected. Within certain cultural contexts, mothers communicated that they were expected to “*just get on with it*” irrespective of the challenges they faced in parenting a disabled child. Seeking support for their health needs might be seen as showing weakness implying you are unable to look after your child. Some participants acknowledged that they may prefer “privacy” and to seek what is familiar within their own cultural experience. It was explained that a religious understanding may encourage the person to “*pray harder*” and seek social support from their “close-knit community” instead of from external programmes. Participants described how reduced knowledge and stigma surrounding childhood disability and mental health, particularly in first generation immigrant populations with low educational status, constrained many parent carers in their community from seeking support. Interpretation of needs could also be restricted by the scope of community languages. For example, it was elucidated that in Somali there are no direct translations for “autism” or “wellbeing,” and so parent carers were working within their community to build an understanding through reinterpretation with familiar concepts.

Ultimately, if the advertised programme was to be perceived as relevant, it needed to be seen as a beneficial and attractive opportunity. Further, social norms of “*being strong*” and “*looking after everybody*,” while potentially seeing aspects of mental wellbeing as “*fluffy*” or preferring independent self-support, remain in male culture.

*“I can care for myself. I can do that on my own. So why do I need someone to tell me about it when I already have … different sources or different platforms where I can get my information instead of, you know, wasting my time on all these programmes going here and there?” (NA16, Black African father)*

Another participant illustrated how intersectionality compounded the issue, highlighting how “*a societal issue [exists] in my community about mental health and [being] a man*” made “*mental wellbeing … even more of a taboo*” and this “*misconception about mental health*” resulted in a “*lack of support for men of our culture*” (A3, British Asian father).

Therefore, explaining the evidential risks to health and mental wellbeing for parent carers through peer word of mouth was suggested as a convincing approach to enlighten the relevant need. For participants who had not attended the HPC programme, being distinctively *peer-led* and focused on parent carers themselves stood out as appealing and crucially meeting a gap.

### Reservations: addressing barriers to readiness to participate in health promotion programmes

Even when the need is recognised and opportunity offered, individuals need to be ready and able to engage with the programme. For parent carers to participate, the timing of the programme needs to coincide with the person’s readiness to engage. This is shaped by social norms and circumstances.

Social circumstances influenced a person’s readiness. Frequently parent carers reported times of being too overwhelmed by their caring responsibilities and in “*fighting*” for their child’s care and education to consider attending a programme for themselves. Providers experienced in working with parent carers with additional social stressors, often in first generation immigrant or other marginalised communities, explained that crises were common, e.g., relating to housing or finances. Therefore, within their hierarchy of needs, parents had less practical and mental/psychological capacity to consider attending a wellbeing programme.

*“… some people are just not in the place at that time when things are being run to be able to have the capacity for it … If they are going through crisis or something is going on where they just cannot focus on anything other than just getting through that … Where there is just nothing left to give to something, that brain space is just not there. Sometimes that can be a big barrier depending on what else is going on in your life. (F28, Somali mother).*

Aware of these social circumstances, some providers had taken steps to meet social needs by providing grocery vouchers at/after each session and indicated that *“if they are dropping out, even after that, that shows you how challenging it is for them [to attend]”* (F2, British Bengali mother).

Other practical considerations were reported to unequally affect certain parent carers. For working parents, often fathers, the day and time of the group was particularly important. It was pointed out that in some cultures where the father’s role was to provide income, the prevailing cost-of-living crisis at the time of this study meant that they were often working longer hours. In some ethnic groups, it was explained that it was uncommon for women to drive and so the venue needed to be local for in-person groups, and partners might need to drive or chaperone them to and from the programme. For single parents, childcare was a major concern for attendance, especially if their child was not in school.

*“… immediately I’m thinking about time. I’m a single mum, and I do not do online, so how am I going to do that.” (NA22, White British mother with additional needs and a child EOTAS).*

Socioemotional reasoning was prominent within parent carers’ reservations about attending. Many highlighted social anxiety and low confidence from their sense of isolation as parent carers, which was accentuated for parent carers of children EOTAS. Understanding and acceptance of the parent carer identity was seen as a critical factor in readiness to attend. Acceptance of the parent carer social identity influenced openness (“*is it for me?*”) to joining with other parent carers in a peer programme. The sense of belongingness within the group was also related to their identity.

*“… mums understand each other, we know how we feel, we know… yeah. All from the community.” (NA12, Somali single mother).*

Fathers were concerned with groups being attended predominantly by mothers: *“how am I gonna feel being the only guy there?”* (A6, White British father, EOTAS), while worrying about their perceptions of fathers.

*“I mean, it can be a bit nerve wracking. It’s not that other parents do not treat you as an equal but there is sometimes that kind of misconception that I may not have that maternal instinct, or I may not understand things like how a mum would.” (A4, White British father).*

Similarly, there were reservations about what such a programme might entail, with fathers concerned it might be “*too heavy*.” Others might feel uncomfortable about sharing, particularly if they were unsure of who was in the group. In seeking to address these barriers and reservations, further efforts were suggested or being undertaken by some providers. In some situations, reservations were simultaneously addressed during word-of-mouth invitations to the programme and other practical support was offered to parent carers, e.g., the EOTAS advocacy group. For some ethnic groups, providers from the same community worked within existing belief structures. In some cases, they explained that deconstruction of unhelpful beliefs around childhood disability and parent mental health was needed to empower parent carers to be self-aware and seek support. Working positively with the strengths of the belief system, the mother’s attendance was framed in a way that it would support her to fulfil in their primary caregiving role more effectively.

*“I honestly believe the lifestyle that a lot of ethnic minority groups lead are often great and it works great because the man takes the role of being the main income provider and the woman takes the role of looking after the household. Those things work because the community really believes those values so we cannot destruct that. That is the core mentality but at the same time while we are doing those duties we are also looking after ourselves so we can achieve those duties even better. I think that is the approach we have to do.” (F36, Bengali mother).*

Strategies suggested for engaging fathers included offering practical activities to bring together male parent carers, overcoming stigma, building confidence and developing a gradual openness towards being part of a group of parent carers.

*“From what I’ve seen with the men’s group … it would be such a progressive thing then. Start with a sort of ‘Dad-to-Dad’ approach, and then say, ‘Well, at this point – when you feel comfortable – we are gonna open it up…” (NA29, White British father).*

Pre-programme engagement also appeared important to parents for rapport building. Facilitators described how they used pre-programme conversations with parents as a key mechanism to addressing reservations. The conversation sets a relational tone of *“accepting without judgement”* while assessing and planning reasonable adjustments in open partnership and allaying anxieties about the programme. This approach is also essential for tailoring programmes to be inclusive.

### Optimisation: tailoring to improve the inclusivity of programme delivery

Several issues were identified for optimising programme delivery to ensure acceptability to the diverse social groups, with varying contexts and preferences.

Group composition was perceived as an important factor. Some mothers from a Muslim background stated that a mixed-gender group would be an immediate barrier to their attendance or would affect their openness if men were present. Some fathers felt that a male-only parent carer group (including facilitators) might “*reduce some of the stigma and make them feel a bit more comfortable to open up*” (A4, White British father). However, many participants (including fathers and Muslim women) felt there were also significant benefits to learning from others in a diverse group. Further, they believed that the shared parent carer identity and attending “*for the same purpose, to access support, provide support, hopefully to learn*” was the most important unifying factor for group cohesion.

*“It’s not about the group has to look like you, or be the same religion, race, or anything else … you have to feel safe, you have to feel comfortable, you have to feel that you are not going to be judged or critiqued when you are going for support and sharing your experiences. That’s not dependent on race.” (NA11, Black British Caribbean single mother).*

Other subtle cultural expectations were explored that might make people comfortable, for example, in how hospitality was provided. It was also suggested that mothers from some ethnic groups *“do not tend to voice”* easily in groups and similarly that fathers were more hesitant about being open. Hence the programme design and facilitation approach were seen as critical with dads *“looking at more the activities that you do”* and not *“necessarily start with talking”* before feeling safe (NA10, White British father).

Parent carers with sensory impairments or neurodiversity expressed they might feel anxious about attending if they felt that their individual needs were not understood and able to be met. Although those with longstanding visual impairment reported feeling confident asserting their needs up front; one participant (NA34, visually impaired mother) highlighted how important it was for facilitators to *“set some time aside … to make [them] aware of any adjustments”* and build a partnership so as they went along they “*could figure it out together.”* Specifically, *“workbooks or any kind of materials … would need the most accessible format”* and examples were given where within groups videos were given verbal descriptions or materials were made into large print for parent carers with visual impairment. One participant also suggested it would help “*if the materials were developed with the neurodivergent in mind as well as the neurotypical*” (A6, neurodivergent White British father of child EOTAS). Facilitators outlined that numerous parent carer attendees on their courses were neurodivergent. This had implications for programme and facilitation adaptation. It was explained that “*because of the openness*” required on programmes, some autistic parents were *“struggling … with understanding what we are doing”* (F31, neurodivergent mother with hearing impairment). Having facilitators with the same lived experience aided these adaptations. Facilitators discussed practices that ensured “*no parent carer [was] left behind*,” *“accepting people on their terms”* and *“suspending judgement”* to create the safe space required.

Facilitators’ mindfulness of cultural issues helped them to emphasise aspects of programme content that might be more meaningful to some communities. The translation of programme materials into different languages was critical for inclusivity of highly represented populations in one urban area. Translation incorporated cultural reinterpretations where necessary, for example, with the concept of wellbeing and programme activities, to enhance relevance and acceptability in some communities.

There was a reflection across the groups that there was a place for both online and in-person delivery to address different barriers related to access. For example, some parent carers *“would not have the luxury to always attend in person”;* online delivery might in particular enable programme attendance of parents of children EOTAS or single parents with their child at home. Parent carers with visual impairment perceived fewer barriers online, compared to attending an unknown venue that would require time to learn to navigate. However, it was noted that online delivery also created barriers, particularly relating to digital literacy and poverty. This is relevant to all parent carers but was considered more likely to affect ethnically diverse parents with intersecting factors of social disadvantage. It was notable that two study participants required the support of their PPI chaperone to set up their video call interviews. Many people highly value social gatherings; for example, participants highlighted “*that face-to-face forum is really important*” for the Somali community.

*“…some of them really like face-to-face, they do not like the online stuff to be honest. Yes face-to-face is clearer to be honest, some of them like their English is very hard. They’d rather just face-to-face.” (NA12, Somali mother).*

Most participants *“prefer the face-to-face meetings, training and activities … rather than the online ones”* (NA9, White European mother of EOTAS child).

*“I do not think it [online] would be as beneficial. You do not show your emotions in the same way when you are at home. My daughters are always home with me so I keep it together in front of them. I am less willing to be vulnerable.” (NA17, White British mother of EOTAS child).*

Across different groups, participants reasoned that in-person attendance enabled greater quality social interaction that supported deeper engagement, hence it would be more *“memorable”* and potentially more impactful.

## Discussion

Our study identifies and builds an understanding of the challenges and barriers that some parent carers might experience to accessing and engaging with parent carer-focused programmes. Within our UK context, we focused on particular underserved parent carer populations: Global Majority communities, fathers, parents of children EOTAS, those with sensory impairment, and identifying as neurodiverse. The identified challenges and strategies that may help improve the access and engagement of some underrepresented groups of parent carers are summarised (Table 3).

A key challenge is to include parent carers who are not, or less, connected within networks, and who have reduced awareness of childhood disability, SEN and how to access support either for their child or themselves. Cultural factors, language barriers, and social norms further complicate access to such programmes not least because these factors constrain some people’s interpretation of their own health needs.

Suggested strategies to improve reach include targeted outreach efforts, community partnerships, and leveraging trusted messengers within underserved communities. The credibility of the messenger advertising a programme is crucial, with trust built through authentic engagement and understanding of community needs. The opportunity for attendance must be presented clearly and resonate with the audience, addressing linguistic, cultural, and social barriers. Strategies to address reservations and cultivating readiness for engaging in a programme include scheduling, addressing socioemotional concerns, and providing practical support. Tailoring delivery for inclusivity involves considering group composition, adapting materials for diverse needs, and alternative options for online and in-person groups. Overall, addressing barriers to access and engagement requires a comprehensive approach, from publicising to programme delivery, tailored to the diverse needs of parent carers in potentially underserved communities. Although we focused on a parent carer programme, similar strategies have been advocated across different types of interventions and populations, indicating that our findings might also be transferrable to other contexts. For example, working with community partners is vital to recognise and align with cultural and religious values in developing sensitive and resonant communication (51). Equally, understanding the specific needs of target populations is critical to create more relevant and impactful health interventions (10).

We acknowledge the broader social determinants of health which affect people’s engagement with health behaviours and programmes (6). A social determinants approach to health behaviours seeks to understand how social factors shape people’s health reflected by *“a dialectic between structure and agency that necessitates situating individuals in context”* [p. 78, (52)], which aligns with our findings. For example, our findings highlight deeply rooted sociocultural norms and beliefs particularly in relation to fathers and some ethnic groups. Social norms within these groups were perceived to constrain support-seeking behaviours amongst parent carers (53). In some cases, this may be explained by a hermeneutic injustice, whereby someone’s social experience obscures their understanding *“owing to prejudicial flaws in shared resources for social interpretation”* [p. 147, (54)]. Participants suggested that the sense of stigma linked to parent carer identity that they felt was related to the degree of awareness and acceptance of disability within their own social groups (53, 55–59). Furthermore, mental wellbeing was identified by fathers and some ethnic groups as a “taboo,” and an added barrier to seeking support. Some HPC facilitators were aware that if parent carers are to have equal opportunities to access programmes, then some beliefs, attitudes and practices that promote wellbeing to a greater extent need to be supported while constraining beliefs challenged. Providers of parent carer health programmes need to be cognisant of existing belief structures to support families in culturally congruent ways, while also empowering them with knowledge and resources to enable support-seeking behaviours and build sustainable resilience for improved outcomes (53, 60–62). Structural inequalities are likely to require multi-pronged community and policy-level strategies to address inequities beyond our suggested approaches linked to advertising and delivering health programmes (9).

In our study fathers theorised that underrepresentation in parent carer health programmes was related to reduced perceived need or that they preferred to meet their wellbeing needs individually. The level of mental health or wellbeing needs in male parent carers compared to mothers has been less evaluated, although it is acknowledged both parents have different experiences and challenges during the caregiving process (21). A survey in Australia reported that 58% of fathers of disabled children reported high depressive symptoms and 61% stress symptoms, as well as low participation in weekly health-promoting activities, with only 26% undertaking solo exercise and 3% social activity (63). Though this was a small sample, it suggests an unmet need in many fathers. Paradoxically, fathers may be reluctant to attend peer-led parent carer group programmes. Therefore, targeted publicising to address reservations and highlight the potential benefits to fathers participating in a health programme might help increase receptiveness and engagement.

Reducing inequity in access and engagement may require adaptations to intervention implementation and delivery while maintaining intervention functions and change mechanisms (64–66). This was evident in facilitators’ views and reported practices in our study. Our study builds on research on parent carer focused programmes by seeking to understand the implementation of such programmes within underserved populations (25). Within the HPC group-based programme, a core intended change mechanism relies on peer support, sharing experiences, and developing a shared social identity of being parent carers who want to improve their health (67). Our findings highlight that personal and social factors (e.g., social disadvantage, gender, ethnicity) might interact with change processes (e.g., feeling safe in the group to share personal experiences). Providers and facilitators need to ensure that these core mechanisms are facilitated when delivering the programme to some underserved groups of parent carers; for example, by drawing on the commonalities of parent carers’ experiences and shared identity. Others have pointed to the importance of meaningful engagement within communities, building trust, fostering long-term relationships, often enacted through third sector organisations, to address inequity (5, 7, 68, 69). In our study delivery partner organisations (as providers) were seen to operationalise their understanding of their community context, including social norms, to adapt their strategies to enhance their reach and build trust with underserved communities to enable improved uptake. Further, facilitators with shared lived experience can adapt programme delivery approach and some materials to be more culturally compatible, which adds to previous knowledge (70). Finally, our findings indicate a nuanced debate around equity considerations with access to online health programmes. A recent framework provides a helpful point of departure for researchers and practitioners to address this challenge at an individual level (71).

### Strengths and limitations

We undertook efforts to involve a diverse PPI group and recruit a diverse sample of participants. To ensure that parents from underserved groups were enabled to participate, we worked with key gatekeepers within different communities and partnered with chaperones during interviews. Our analysis and interpretation were challenged and strengthened by the critical contributions and triangulation with the PPI group and other stakeholders.

By interviewing numerous parent carers with no experience and those with experience of parent carer-focused health programmes, we were able to explore a wider variety of perspectives relating to access relevant to the target underserved groups. In addition, parent carers from diverse backgrounds, as facilitators, shared their strategies and successes of promoting and delivering the programme equitably in their community with the authority of lived experience.

Within our sample, participants had multiple intersecting characteristics, so it was not always feasible or sensible to ascribe particular characteristics (e.g., being a father and/or ethnically diverse) directly to specific barriers to access/engagement. Despite some differences in how parent carers with similar demographics might experience inequity, we found that participants described many more common barriers and challenges. Hence our findings were presented for the whole sample with specific examples noted to provide context. While our sample could not be fully exhaustive of all permutations of diversity and intersectionality, we nevertheless did include a range of perspectives from our targeted social groups.

Education is seen as “a fundamental social determinant of health - an upstream cause of health” (72). The educational status in our sample, which leant towards more highly educated individuals, may have reduced the diversity of perspectives overall. This meant that certain participants from Global Majority backgrounds, who were second-generation immigrants with higher education, offered their insights on individuals in their communities, typically first-generation immigrants with lower socioeconomic and educational status, often adhering to more “traditional religious” beliefs and norms. There is a danger that assessing individual or family beliefs and practices could become ethnocentric if comparisons are made against untested normative assumptions from researchers without shared cultural experience. While it is impossible to fully understand the lived experience of many of the participants, nevertheless we remained cognisant and empathetic with an open approach that sought to learn from their perspectives and sense-checked these with our PPI and stakeholder groups.

## Conclusion

Addressing barriers to parent carers’ equitable access to and engagement with health and wellbeing programmes requires a comprehensive, multifaceted approach, from initial outreach to delivery, tailored to be mindful of the diverse needs of parent carers in our underserved communities. The identified strategies to help address these barriers, although focused on parent carer programmes, are likely to be applicable to a wider range of health behaviour change interventions.

## Data Availability

The datasets presented in this article are not readily available because consent was not obtained from participants to share the anonymised data set with other research groups. Requests to access the datasets should be directed to christopher.morris@exeter.ac.uk.
