# Supplementary material for: Ensuring equitable access, engagement and ability of socially and ethnically diverse participants to benefit from health promotion programmes: a qualitative study with parent carers of disabled children
Source: Front Public Health. 2024 Sep 30;12:1445879. doi: 10.3389/fpubh.2024.1445879 (PMC11472851; doi:10.3389/fpubh.2024.1445879)
Supplement: Supplementary file 1 [file Table_1.pdf]

Supplementary document

Interview topic guide

n.b. The topic guide was adapted according to whether the participant had experience as an attendee, non-attendee or facilitator on the Healthy Parent Carer programme

Table 4: Topic guide

**1. Experience of parent carer focused programmes**<br>a. Have you had experience of the Healthy Parent Carer programme or any other structured programmes focused on parent carers? (prompt for details of other programmes if attended)<br>b. Was there any focus on your health and wellbeing within that programme?
**2. Views and experiences related to access and recruitment**<br>a. Where and how might you find out about such programme for parent carers?<br>i. Where do/might people from your community (go to) find out about parent carer programmes?<br>b. How could we make sure that different parent carers find out about programmes or similar support for them?
**3. What barriers might there be, if any, to people from your community thinking about taking part in this type of programme?**
**4. Views and experiences related to format and engagement**<br>a. What do you think about the programme being delivered in groups online using Zoom or in person?<br>i. What might make it difficult for parent carers to engage with the programme online?<br>ii. Do you have solutions to these barriers?
**5. Views and experiences related to content and delivery.**<br>a. The HPC covers different topics related to promoting health and wellbeing through interactive discussions and activities.<br>b. Do you have any thoughts on what would be particularly engaging for other parents [of shared background]?
**6. Views and experiences related to group composition**, e.g.:<br>a. Do you have views on what the make-up of the groups should be?
**7. Summary**<br>a. Do you have any other thoughts or suggestions on how to make programmes for parent carers more accessible (or inviting / acceptable) and engaging to different groups [group they represent] of parent carers?<br>b. Is there anything else you'd like to say that we haven't covered?
